# Comprehensive evaluation of bioactive properties and metabolomic profiling of probiotic bacteria *Lactococcus lactis* (MKL8)

**DOI:** 10.1038/s41598-025-02327-x

**Published:** 2025-07-01

**Authors:** Tholla Madana Shivani, Mythili Sathiavelu

**Affiliations:** School of Biosciences and Technology, Vellore Institute of Technology, Vellore, 632014 India

**Keywords:** *Lactococcus lactis*, Anti-oxidant, Anti-diabetic, Anti-inflammatory, Secondary metabolites, Biofilm, Microbiology, Biofilms

## Abstract

This study evaluated the bioactivity and the metabolomics of *Lactococcus lactis* MKL-8, a probiotic bacterium that was isolated from *Murraya koenigii*; contributing to broadening the diverse ecological diversity of *Lactococcus lactis*. The primary objective of this study was to explore the multifunctionality of MKL-8 by bringing together its bioactive potential and its metabolomic profile to assess its suitability for probiotic use. Identification of probiotic strains demonstrating multifunctional therapeutic properties along with proven safety is the most significant challenge in meeting increasing demand for natural therapeutic substitutes to synthetic drugs. MKL-8 showed high antioxidant activity with 90.19% inhibition, exhibits an anti-diabetic effect with 60% inhibition of α-amylase, and anti-inflammatory activity with 99% inhibition of protein denaturation, demonstrating significant potential as a natural therapeutic agent. The strain also exhibited significantly high ability to form biofilm and synthesized a considerable amount of Exopolysaccharides (EPS). GC-MS analysis revealed bioactive metabolites which are reported in the literature to have, immunomodulatory effects, antioxidant, collagen synthesis, and wound healing properties. The presence of these compounds was further affirmed by FT-IR spectroscopy, further indicating the importance of MKL-8 as a multifunctional probiotic. This paper presents the isolation and versatile applications of MKL-8, demonstrating it is a valuable natural probiotic strain. Collectively, these findings establish MKL-8 having potential health benefits making it a promising choice for progressive probiotic applications both in the healthcare industry and the functional food sector.

## Introduction

Lactic acid bacteria (LAB) are one of the biggest contributors to human gut microbiota, which plays a profound role in maintaining health. It synthesizes antimicrobial compounds in situ, modulates the balance of Gastro Intestinal (GI) commensal bacteria, and deprives invader pathogens of nutrients for their growth, thus exhibiting a defensive action within the GI tract. Among the various organisms being used as probiotics, lactic acid bacteria are the most frequently used, which is part of the gastrointestinal native flora and recognized as safe organisms by the Food and Drug Administration (FDA)^1^. It refers to a class of facultatively aerobic, non-sporulating, gram-positive, anaerobic cocci or rods that produce lactic acid^2^. Most probiotic strains belong to the genera *Bifidobacterium* or *Lactobacillus.* This led us to study *Lactococcus lactis*, another important LAB that has been demonstrated to exhibit promising probiotic characteristics. Research has highlighted the different characteristics of *Lactococcus lactis* strains that possess several features of probiotics. Since *Lactococcus lactis* has been utilized in the past for food fermentation, it is considered a “Generally Recognized as Safe or GRAS.” Due to its biocompatibility and ability to release drugs in targeted tissues, it has also been considered a delivery system of therapeutic molecules in the stomach and intestines. Although numerous studies documented *L. lactis* as a GRAS organism, the possible novelty of the strains from non-dairy, plant-derived sources has not been clearly explored. Also, the bioactivity perspective of such strains with regard to its antioxidant, anti-diabetic, and anti-inflammatory capacities along with its secondary metabolites has not been sufficiently elaborated yet. The same case applies to strains from medicinal plants such as curry leaves where the understanding of these functional enhancers and ability of probiotics to utilise them has not been fully researched. This research intends to fill this gap by investigating the therapeutic potential of the *Lactococcus lactis* MKL-8 isolated from curry leaves and its inherent capacity to produce secondary metabolites that provide huge health benefits along with its bioactive properties that have the potential to alter the gut flora^3^.

A critical aspect of probiotic efficacy lies in their diverse bioactive properties. LAB, including *Lactococcus lactis*, contributes to health by triggering immune responses and inhibiting pathogen growth through several mechanisms. Several studies have indicated the inhibition of α-glucosidase, an enzyme needed to monitor blood sugar for diabetic patients^4^. The LAB also synthesize bacteriocins, which are protein molecules with antagonistic effect on pathogenic bacteria thus, adding to the antimicrobial effect. For example, Hernández-González et al., examined the antibacterial, probiotic, and immunomodulatory effects of LAB in veterinary medicine, while ascribing its effects to an improvement of immune response and an effective modulation of the gut flora^5^. In the same respect, the probiotics have shown antagonistic action against both the Gram-negative and Gram-positive pathogenic bacteria and that antagonism activity provides a compelling rationale for use of the probiotics as the potential substitute to the antibiotics needed to combat the growing problem of antibiotic resistance and to promote the healthy colonisation of the gut^6^.

A recent WHO survey revealed that 70 to 80% of the global population uses alternate complementary medicine mainly originated from herbs, and their demand is gradually increasing. Hence, one can use relatively cheap and safe products like probiotics to manage such issues^7^. Chen et al., in their study conducted in 2020 gauged the safety of strains of *Lactococcus lactis* and *Leuconostoc lactis*. They also went further to perform in vitro experiments to study the antibacterial efficacy, surface characterization, heat stability, resistance to antibiotics, and tolerance to acids and bile of the strains^8^.

The bioactivity of probiotics is often linked to the production of secondary metabolites, which are bioactive compounds that can exert significant therapeutic effects. Some *Lactococcus* species, including *Lactococcus lactis*, have gained importance by playing major role in production of food products and medical necessities^9^. Further, these bacteria possess an ability to produce strong bioactive compounds, peptides, proteins, and live cells to improve the digestion systems, immune system agents, and protection against heart diseases, diabetes, advanced glycation end products, and the neutralization of mycotoxin in foods and feeds^10^. Furthermore, LAB-derived useful compounds can be recovered for its use in applications that produce antimicrobial peptides, bacteriocins, and enzymes which assist in improving food preservation, drugs production, and disease prevention. These compounds possess significant therapeutic uses in the medical and industrial field which makes LAB a valuable resource for biotechnology.

In our previous work, we were able to isolate and identify *Lactococcus lactis* from curry leaves, a plant with medicinal importance and rich in phytochemicals^11^. This preliminary study provided the background for investigation into *Lactococcus lactis* as a non-dairy, plant-sourced probiotic. Although that study is mainly concerned with the isolation and initial characterization of this strain, the present work is intended to extend beyond the basic functions and explore the functional characteristics of this strain. More specifically, the present work will focus on the bioactivity of *Lactococcus lactis*, which encompasses diabetic modulatory effects, anti-inflammatory, anti-oxidant effects, biofilm-forming ability, and gelatinase production. Moreover, the identification of the secondary metabolites synthesized by this strain is done in order to give a deeper understanding of the working principles of this health promoting strain and its therapeutic values.

## Materials and methods

### Isolation, characterization and molecular identification of lab

In a previous study, *Lactococcus lactis* (MKL8) (Accession number OR342073) which was isolated from *Murraya koenigii*, was characterized for its probiotic properties^11^. The strain showed a very good tolerance to GI conditions such as high concentrations of bile and low pH and a good ability to adhere to Caco-2 cells. MKL8 also exhibited improved hydrophobicity, auto-aggregation properties and showed antibacterial activity against different pathogens without showing any toxicity as determined through MTT and hemolysis assays. Based on these observations, the present work is aimed at assessing the bioactivity of MKL8 in addition to, determining the secondary metabolites. This study aims to further elucidate the functional properties of MKL8, thus extending the possible use of this protein in creating novel health-promoting foods.

### In vitro bioactivity assessment of MKL8

#### Antioxidant activity

MKL8 for its antioxidant activity was analysed through 2, 2-diphenylpicrylhydrazyl (DPPH) radical scavenging method according to Ramalho, J.B. et al., with minor modification^12^. 0.003 g of DPPH was dissolved 100 mL of methanol to prepare 0.5 mM DPPH. A single colony of the MKL8 was transferred from the plate into 5 mL MRS broth and incubated in shaker at 25 °C for 5 days. The culture was centrifuged at 3000 x g, 10 min at a temperature of 4 °C, with the result being the supernatant. For the assay, an aliquot of the supernatant in methanol was combined with 2.5 mL of the 0.5 mM methanolic DPPH solution. It was then incubated in complete darkness for 30 min at 37 °C. The optical density (OD) of samples was determined using a UV-visible spectrophotometer (SHIMADZU UV-1280) at a wavelength of 517 nm, with ascorbic acid being used as a positive control and methanol being the negative control. Triplicates were maintained throughout the experiment. The DPPH radical scavenging activity was calculated using the below formula:

$$\% {\text{ inhibition }}=[({\text{Abs of Control}}\, - \,{\text{Abs of test}})/{\text{Abs of Control}}] \times {\text{1}}00$$

#### Antidiabetic activity

The α-amylase enzyme inhibition assay was performed to assess anti-diabetic potential of MKL8^13^. For the assay, 500 µl phosphate buffer with concentration of 0.02 M and a pH of 7 was prepared to which 500 µl of 1 mg/mL α-amylase enzyme solution was added with a varying concentration of test sample and the standard drug Acarbose. The mixture was allowed to stand at 37 °C for 10 min prior the incubation. Afterwards, 10µL of 1% soluble starch solution was added to all mixtures and incubated at 37 °C. Finally, 100 µL of 1% iodine and 5 mL of distilled water were added to stop the reaction in each tube. The absorbance of final solution is determined at 565 nm with the help of a UV-visible spectrophotometer. To rule out the influence of the media on enzyme activity, MRS media (where *Lactococcus lactis* was grown) was included as a control along with the control obtained from the enzyme solution containing starch and buffer but devoid of the inhibitor so as to correct for non-specific absorbance. The % of inhibition of α-amylase was calculated using the following formula:

$$\% {\text{ Inhibition }}={\text{ }}[({\text{Abs of Control}}\, - \,{\text{Abs of test}})/{\text{Abs of Control}}] \times {\text{1}}00$$

The assay was performed three times, and the mean of the result was used in the evaluation.

#### Anti-inflammatory activity assay

To determine the effect of the MKL8 on the anti-inflammatory effect, the albumin denaturation inhibition assay was performed using Gunathilake et al., procedure with few modifications^14^. A pure culture of MKL8 was streaked onto MRS broth and allowed to grow under shaking conditions at 25 °C for five days. Following incubation, the culture was then centrifuged at 3000 × g at 4 °C for 10 min, and clear supernatant was harvested.

For the assay, 4.780 mL of phosphate buffered saline (PBS, pH 6.4), 0.2 mL 1% Bovine Serum Albumin (BSA), and different volume of sample was mixed gently. The reaction mixture was allowed to stand for 20 min at 37 °C, allowing the target proteins to become active, and later was subjected to heat at 57 °C for 5 min to denature the proteins. The absorbance of the solution was then determined through the use of a UV-visible spectrophotometer after the solution had cooled. The control was phosphate buffer solution with no sample added to it. Dexamethasone was employed as the standard control to check the efficiency of the assay. All test incubations were performed in triplicate. The % inhibition of protein denaturation was calculated by:

$$\% {\text{ inhibition of protein denaturation}}\,=\,{\text{1}}00 \times ({\text{1}}\, - \,{\text{A2}}/{\text{A1}})$$

where, A1 = Absorption of the control, A2 = Absorption of test.

#### Biofilm formation assay

The ability to form biofilm is critical for probiotic bacteria, as it strengthens their attachment properties to the gut epithelium and the ability to prevent pathogenic colonization. In this study, both qualitative and quantitative characterization was done to determine the biofilm-forming capacity of MKL8^15,16^.

The qualitative assessment entailed inoculating 2% MKL8 into sterile MRS broth in a test tube and incubating for 48 h at 30 °C in orbital shaker at 130 rpm. *Staphylococcus aureus* was used as the control. After incubation, the tubes were washed using 0.1 M PBS, and stained with 1% crystal violet for 30 min and then proceeded to rinse with distilled water. Biofilm formation was confirmed by the appearance of violet coloration on the walls of the test tube.

For quantification purpose, 200µL of MKL8 was dispensed into 3 wells of 96 well plate. Blank well had media only, while control wells were filled with media mixed with the respective inoculum – *S. aureus* in this case. After static incubation for 48 h at 37 °C, the wells were gently rinsed using PBS for removal of any free-floating bacterial cells. The cells adhered to the walls were fixed using 200µL chilled methanol and afterwards washed using distilled water before being stained with 0.1% crystal violet for 30 min. Excessive stain was rinsed with tap water, and bound stain was eluted by using 250 µL of glacial acetic acid (33% v/v). The absorbance was determined at 595 by using a microplate reader. MKL8 was considered a strong biofilm producer if its OD values were either equal to or greater than that of the positive control and showed high biofilm production, which is desirable for probiotics.

#### Production, purification and quantification of exopolysaccharides

For EPS extraction, 50 mL of MRS broth containing bacterial culture was incubated at 37 °C for 48 h at 120 rpm. Subsequently, the culture was centrifuged at 8,000 × g at 4 °C for 15 min to obtain bacterial pellet. The proteins in the supernatant were precipitated with cold 12% w/v trichloroacetic acid and then centrifuged again at 8,000 × g at 4 °C for 20 min. Finally, EPS was precipitated out of the supernatant by adding 2 volumes of cold ethanol to the culture and incubated at 4 °C. Following the second centrifugation of 2,500 × g for 20 min, the precipitate was gently resuspended in distilled water, reprecipitated by adding an equal volume of cold ethanol and washed again with distilled water. After dialysis, the final pellet obtained was washed, and total carbohydrate content was estimated by employing phenol-sulfuric acid reagent protocol using the standard as D-Glucose^17,18^.

#### Gelatinase hydrolysis assay

Evaluating gelatinase activity is important to determine the proteolytic potential of MKL8 that may affect its characteristics in functional probiotics. To assess this, Luria–Bertani medium agar (1.5%) was prepared with 3% gelatin, and the pH was brought to 7.2, and MKL8 were spot inoculated on the surface of agar followed by incubating for 72 h at 37 °C. The isolate was spot-inoculated on the solid medium and incubated at 37 °C for 72 h. Plates were monitored for the growth before being flooded with 10 mL of Frazier’s solution (20 mL HCl (37% v/v), 15.0 g HgCl₂ and 100 mL of distilled water) to precipitate all the unhydrolyzed gelatin. The appearance of a distinct halo around the colonies was employed as a means of confirming the presence of gelatinase due to the hydrolysis of gelatin^19^.

### Extraction and identification of secondary metabolies

#### Extraction of metabolite by liquid-liquid separation

For the extraction and concentration of the active metabolites from the cell-free supernatant (CFS) of the isolated probiotic bacteria, a liquid-liquid extraction process was used. An equal volume of chloroform was added to a 200 mL volume of CFS of MKL8, and the two solutions were shaken in a separatory funnel. The extraction was performed three times and, the mixture was first left to settle at room temperature so as to give clear layers. The chloroform layer, which contained the metabolites, was collected and concentrated using a rotary evaporator (Model: RE100-Pro). The dried extracts were then stored at -20 °C for further analysis^20^.

#### Bacterial metabolite profiling via gas chromatography–mass spectrometry (GC-MS)

Metabolites profiling of MKL8 was done using GC-MS at VIT-SIF Lab, Division of Chemistry. Perkin Elmer Clarus 680 GC system equipped with a Clarus 680 EZ Mass Spectrometer was used to perform GC-MS analysis. Chromatographic separation was achieved on fused silica column coated with Elite-5MS (95% dimethylpolysiloxane; 5% biphenyl, dimensions: 30 m × 0.25 mm internal diameter (ID) × 250 micrometres dual frit). Helium was used as the carrier gas for the chromatographic separation process, while the flow rate was maintained constant at 1 mL/minute. The injector temperature was maintained at 260 °C in the course of the analysis. Temperature profile of the oven was ramped up from 60 °C to 300 °C at the rate of 10 °C/minute and was held at 300 °C for 6 more minutes. Mass spectra were taken at 70 eV and, scanning time was 0.2 s and scan interval of 0.1 s. The acquired spectra were further analysed and matched against the NIST (2008) database with the help of TurboMass Software version 5.4.2 (PerkinElmer, Waltham, MA https://www.perkinelmer.com) for compound identification^21,22^.

#### Characterization of functional groups using fourier transform–infrared (FT-IR)

Functional groups of extracted metabolites was identified using Fourier Transform Infrared Spectroscopy (FT-IR) with a Nicolet iS50 from Thermo Scientific, USA. The FT-IR analysis was conducted on the dried crude extract of MKL8. Characteristic absorption within the 4000 to 500 cm^−1^ range was used in the identification of functional groups present in the sample. The obtained infrared spectra were taken from the absorption of infrared light at certain frequency ranges, which in turn represent the characteristic vibrational frequencies of chemical bonds in the extract. The obtained spectra were referenced against standard IR spectra to determine the functional groups present^23^.

### Statistical analysis

All the data analysis were done using GraphPad Prism version 10.0.2 (GraphPad Software, San Diego, CA; https://www.graphpad.com). software. For data comparison, one-way ANOVA followed by Tukey’s HSD post-hoc test was done. Statistical differences were considered significant at a p-value less than 0.05 (*p* < 0.05).

## Results

### Isolation, characterization and molecular identification of lab

*Lactococcus lactis* (MKL8) (GenBank accession no: OR342073 isolated from *Murraya koenigii* demonstrated high survival in low pH and high bile concentration present in the human gastrointestinal tract. It also exhibited adhesion ability to Caco-2 cells, which implies a reasonable colonization ability in the gut. Functional characteristics were confirmed by surface hydrophobicity and auto-aggregation profile. In addition, antibacterial assay confirmed that the MKL8 inhibited a wide range of pathogenic bacteria. Safety assessments including MTT cytotoxicity and hemolysis assays supported the findings that the strain is non-toxic. Molecular identification confirmed the strain to be *Lactococcus lactis*.

### In vitro bioactivity assessment of MKL8

#### Anti oxidant activity

**Fig. 1:**
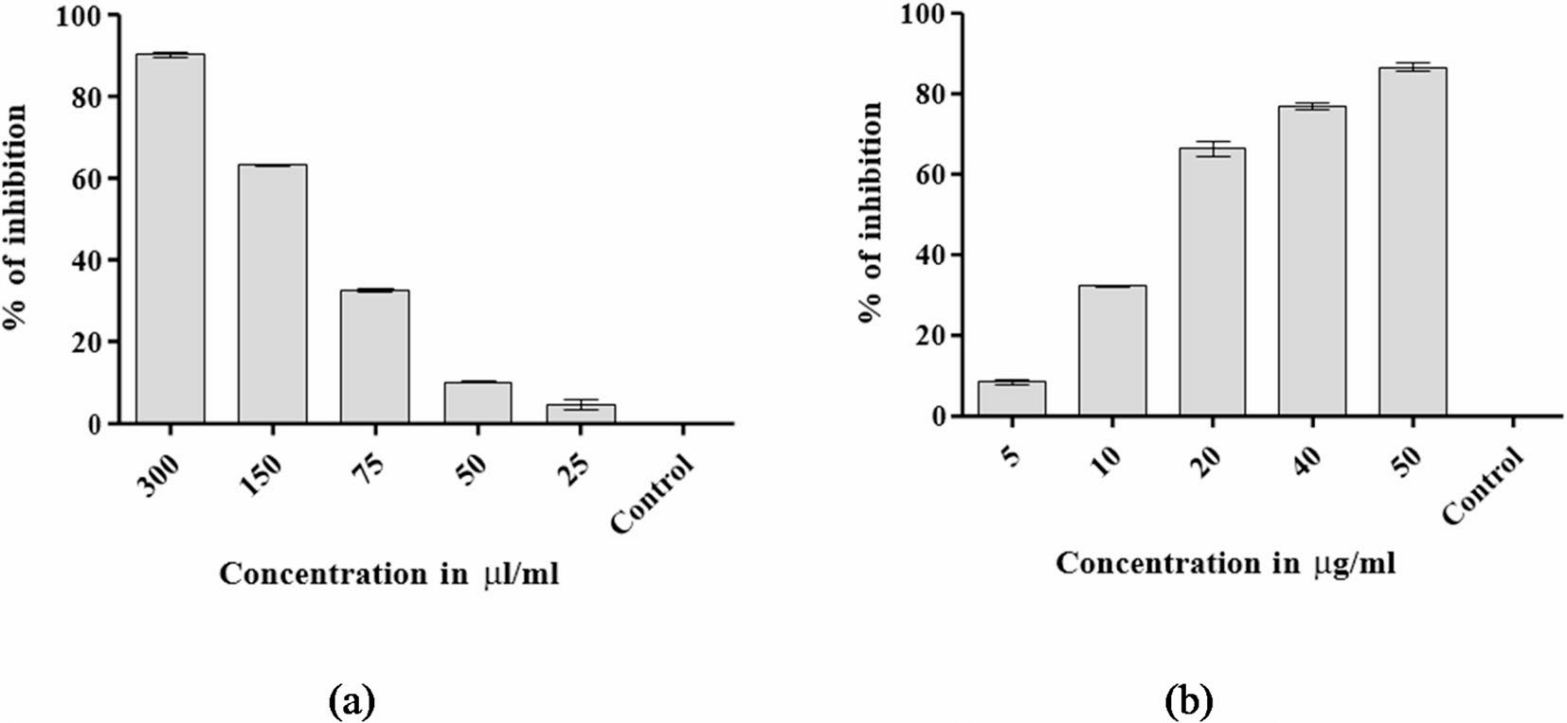
Graphical representation of antioxidant activity of (**a**) MKL8 and (**b**) Ascorbic acid showing percentage inhibition at various concentrations. Data are expressed as the mean ± standard deviation of three replicates (*n* = 3); *p* < 0.0001.

Antioxidant activity is one of the primary features of probiotics as it is held accountable for mitigating oxidative stress in the host organism^24^. Antioxidant activity study revealed that MKL-8 demonstrated concentration-dependent antioxidant activity. The concentration of MKL-8 used in the antioxidant test ranged from 25 µL/mL to 300 µL/mL, while the positive control (ascorbic acid) ranged from 5 µg/mL to 50 µg/mL. Notably, at the highest concentration of 300 µL/mL, MKL-8 demonstrated 90.19% free radical inhibition, indicative of superior antioxidant activity.The value was in extremely close proximity to that of the positive control, ascorbic acid, with 86.57% inhibition in a 50 µg/mL concentration (Fig. 1). One-way ANOVA statistical analysis also established that there existed an extremely highly significant difference (*p* < 0.0001) among the test concentrations. Tukey’s HSD post-hoc test revealed significant difference between the majority of the concentration pairs again proving MKL-8’s dose-dependent antioxidant activity. The percentage inhibition decreased with decreasing concentrations of MKL-8, specifically to 63.15% at 150 µL/mL, 32.58% at 75 µL/mL, 10.17% at 50 µL/mL, and 4.69% at 25 µL/mL. The trend was identical in ascorbic acid, where inhibition decreased from 86.57% at 50 µg/mL to 8.42% at 5 µg/mL. These results are consistent with the fact that MKL-8 exhibits high antioxidant activity at higher concentrations and is a possible natural antioxidant.

#### Anti diabetic activity

The percentage inhibition of α-amylase activities by the treatments of MKL-8 and the standard drug Acarbose indicated a significant level of inhibition, with 87.00%, at the highest concentration of 50 µg/mL. Acarbose which is a well-known inhibitor, was more effective at this concentration, but MKL-8 also had a considerable inhibition value of 60% at 50 µg/mL while showing a possible applicability as a natural, probiotic substitute for the regulation of α-amylase activity. A one-way ANOVA revealed that there was statistically significant difference among the groups (F = 31086.14, *p* < 0.001). Post-hoc analysis using Tukey’s HSD test further validated significant difference between the majority of the groups (*p* < 0.05), establishing that although Acarbose inhibited better, MKL-8 also showed significant inhibition. The level of significant inhibition achieved, especially at higher concentration, indicates the effectiveness of MKL-8 in being part of anti-diabetic studies as a natural α-amylase inhibitor.

#### Anti-inflammatory activity assay

In this study, MKL-8’s anti-inflammatory ability was tested in different concentrations. At the highest concentration of 300 µL/mL, MKL-8 demonstrated a more profound protective effect against protein denaturation the percentage of inhibition being 99%. This activity is highly significant compared to the positive control, Dexamethasone, which had 83% inhibition. There were no significant differences between the triplicate trials of MKL-8 and Dexamethasone using One-way ANOVA (*p* = 0.999). The post-hoc Tukey’s HSD test revealed no statistically significant difference (*p* > 0.05) between concentration groups for both compounds, despite observing higher inhibition with increasing concentration. The results are presented in more detail in Table 1. These results indicate that an improved anti-inflammatory potential of MKL-8 is observed at the higher concentration which makes it a potential molecule for anti-inflammatory use.

**Table 1:** Anti-inflammatory activity of MKL8 and dexamethasone at various concentrations.

Concentration	Dexamethasone% inhibition	Dexamethasone confidence interval (CI)	MKL-8% inhibition	MKL-8 confidence interval (CI)
6.25 µg/mL	1.84 ± 0.78	1.84 ± 1.49	–	
12.5 µg/ mL	2.99 ± 0.75	2.99 ± 1.45	–	
25 µg/ mL	23.46 ± 0.96	23.46 ± 1.88		
50 µg/ mL	67.03 ± 1.77	67.03 ± 3.45		
100 µg/ mL	83.23 ± 1.04	83.23 ± 2.04	–	
25 µl/ mL	–		5.54 ± 0.75	5.54 ± 1.38
50 µl/ mL	–		26.97 ± 0.58	26.97 ± 1.12
75 µl/ mL	–		50.95 ± 0.65	50.95 ± 1.26
150 µl/ mL	–		73.89 ± 1.03	73.89 ± 2.00
300 µl/ mL	–		99.21 ± 0.01	99.21 ± 0.02
Control	0.00 ± 0.00		0.00 ± 0.00	0.00 ± 0.00

### Biofilm formation assay

Biofilm formation potential of MKL8 was assessed both quantitatively and quantitatively.

Qualitative assessment through crystal violet staining demonstrated the ability of biofilm formation as evidenced by purple staining at the walls of the test tubes. In quantitative analyses, using the microtiter plate assay, the mean OD of MKL8 was 1.14 ± 0.04, which was slightly less than the positive control, *Staphylococcus aureus*, with a mean absorption of 1.20 ± 0.05. Still, MKL8 possessed a high biofilm-forming ability that can serve as evidence for its ability to adhere to the intestinal mucosa and prevent gut pathogens from colonization.

### Production, purification, and quantification of exopolysaccharides

EPS are significant in the formation of biofilm in the prospective probiotics because they give the foundation for microbial attachment to any surface. This makes the probiotics to be more stable and persistent in the gut, hence increasing their ability to promote gut health. Exopolysaccharides (EPS) from MKL8 was produced, purified, and quantified. The final yield of crude EPS was 55 mg, and the concentration of polysaccharide in the purified EPS was determined to be 108.35 µg/mL using the phenol-sulfuric acid method. The quantities for all of these parameters demonstrate that MKL8 produces a significant level of EPS, which suggests its ability to contribute to biofilm formation as well as probiotic activity.

### Gelatinase hydrolysis assay

**Fig. 2:**
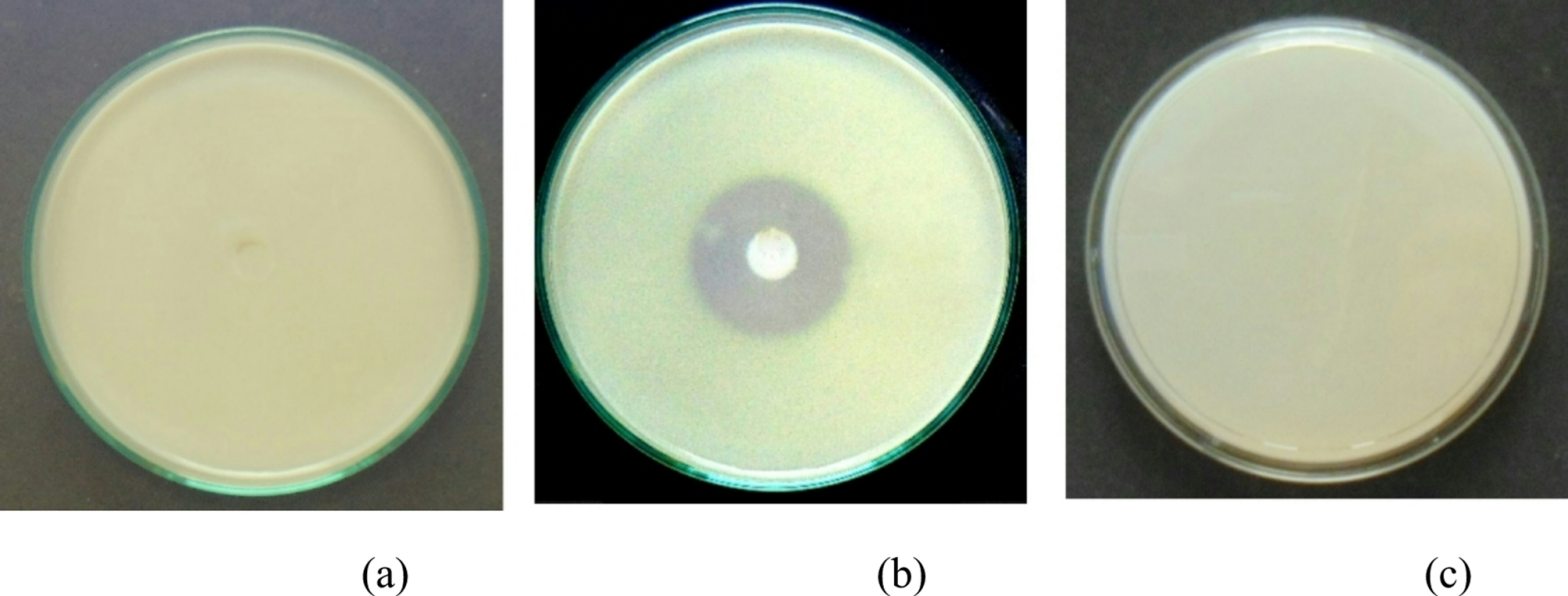
Gelatinase production by (**a**) MKL8 (**b**) *Enterococcus faecalis* (**c**) Control.

The gelatinase production assay showed that the MKL-8 did not produce gelatinase because no clear zones appeared on the gelatin plates (Fig. 2). This result was similar to the negative control in which no gelatinase activity was observed. On the other hand, the positive control, *Enterococcus faecalis* (MTCC 2729), showed a distinct zone of gelatinase activity. Since gelatinase is a significant virulence factor, the absence of gelatinase activity in MKL-8 may indicate its non-pathogenic characteristic, which supports the biopsychosocial consideration in the potential use as a safe probiotic.

### Profiling of bacterial metabolites via gas chromatography – mass spectrometry (GC-MS)

**Fig. 3:**
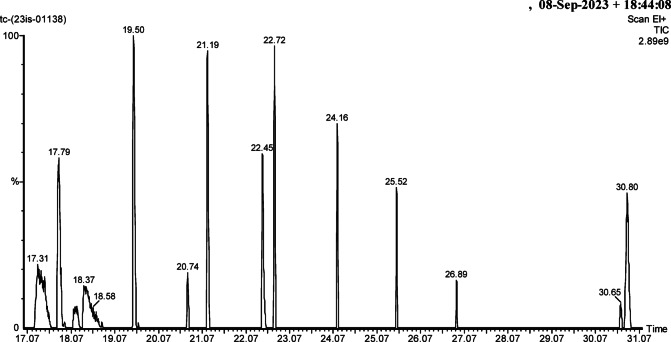
GC-MS chromatogram for MKL8.

GC-MS analysis of the crude metabolite extract of MKL8 after using chloroform as extraction solvent revealed the presence of several important bioactive secondary metabolites. Oleic acid was identified, which is known for its immunomodulating ability and capacity to prevent cellular damage, induced by inflammation and oxidative stress. Also, the dipeptide Glycyl-L-Proline was found, considered to affect collagen synthesis, and skin wound healing processes. The chromatogram of the extracts is shown in Fig. 3. These results reveal that MKL8 is rich in various bioactive biomolecules and holds promise as a probiotic. The list of the identified metabolites and their associated biological activities is summarized in Table 2 below.

**Table 2:** List of chemical compounds identified using GC-MS from MKL8 crude extract.

RT	Compound name	Molecular formula	Molecular weight	Structure	Uses	References
25.52	3-Azonia-5-Hexene-1-ol, N,N-Dimethyl-, Carbamate Ester, Bromide	C_8_H_17_N_2_O_2_+	173		Antimicrobial properties	^25^
22.72	Dichloroacetic acid	C_11_H_20_O_2_C_l2_	254		Potential anticancer properties	^26^
21.19	9-Eicosene, (E)	C_20_H_40_	280		Medicinal properties, including hypotensive effects on blood pressure, and exhibit antimicrobial activity, potentially attributed to its hydroxyanisole content, Cytotoxic properties	^27^
20.74	Oleic Acid	C₁₈H₃₄O₂	282.47		Anti-inflammatory, antioxidant, and cardiovascular benefits	^28^
18.37	2,5-Cyclohexadien-1-One, 3,5-Dihydroxy-4,4-Dimethyl-	C_10_H_12_O_4_.	196.2		Potential antioxidant properties	^29^
17.31	3-(1,3,6-Trimethyl-4-Oxo -3-Piperidinyl)Propanenitrile	C_11_H_18_N_2_O	194		Neuroactive and anti-inflammatory properties, potentially be investigated for neuroprotective effects or as a lead in drug discovery	^30^
17.31	Glycyl-L-Proline	C_7_H_12_O_3_N_2_	172		Glycyl-L-Proline has potential applications in improving skin health due to its role in collagen synthesis. It may also have benefits in wound healing and as a component in dietary supplements aimed at joint and skin health.	^31^

### Characterization of functional groups using fourier transform–infrared (FT-IR)

**Fig. 4:**
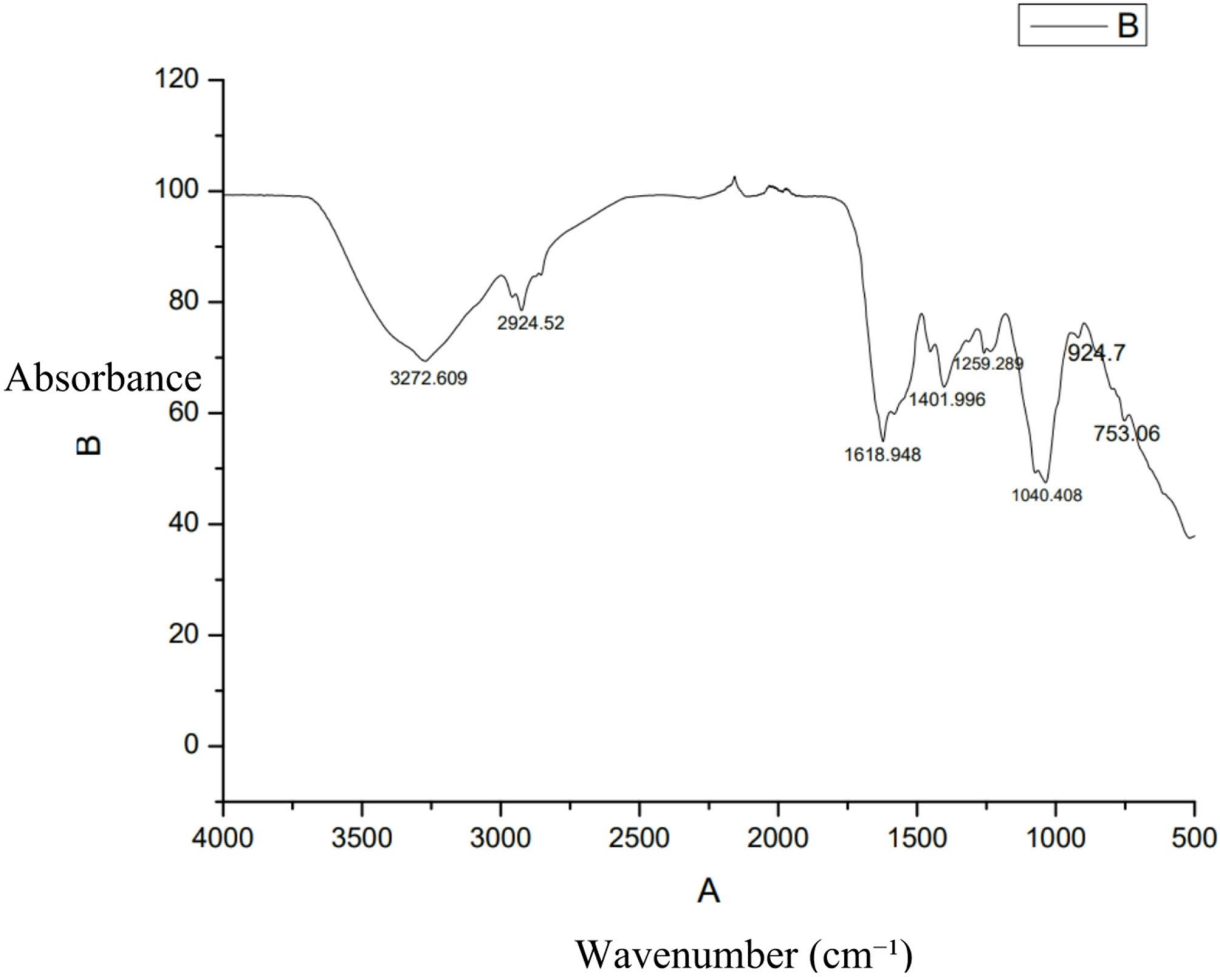
FTIR spectrum of MKL8 with solvent chloroform.

The FT-IR spectrum of MKL8 shows definite peaks for various functional groups that are in correspondence to the compounds identified in the GC-MS study. A highly resolved and wide peak at 3272.609 cm^−1^ is associated with the O–H stretching of phenolic or hydroxyl groups; 3-Azonia-5-Hexene-1-ol, N,N-Dimethyl-, Carbamate Ester, Bromide as well as Oleic Acid. The peaks at 2924.52 cm^−1^ were attributed to the C-H stretching vibrations typical of alkanes, were associated with 9-Eicosene, (E) and Oleic Acid. The spectrum also exhibited intense bands at 1618.948 cm^−1^, 1401.996 cm^−1^, and 1259.289 cm^−1^ which represents C = C stretching (linked to 9-Eicosene, (E) and Oleic Acid), C-H bending (associated with 3-Azonia-5-Hexene-1-ol, N,N-Dimethyl-, Carbamate Ester, Bromide and Oleic Acid), and C-N stretching or C = O stretching (related to 3-(1,3,6-Trimethyl-4-Oxo-3-Piperidinyl)Propanenitrile and Glycyl-L-Proline), respectively. Moreover, another high intensity peak is observed at 1040.408 cm^−1^ which attributed the C-O stretching of Dichloroacetic Acid and the peak at 924.7 cm^−1^ attributed to the out of plane C-H bending of the 3-Azonia-5-Hexene-1-ol, N,N-Dimethyl-, Carbamate Ester, Bromide. These functional groups are related to the compounds identified in the GC-MS analysis, as well as to the chemical composition of the extract and metabolic products with biological action. The FTIR spectrum is presented in the Fig. 4 below.

## Discussion

The *Lactococcus lactis* strain used in this research from *Murraya koenigii* (curry leaf) possesses profound probiotic and bioactivity characteristics, which supports the findings and solutions on the capabilities of this bacterium. This strain, isolated from an unconventional source adds a novel dimension into the probiotic potential of *Lactococcus lactis*, as such sources are typically underexplored in the search for novel probiotics. It is therefore worth to further investigate the unique properties of Lactococcus lactis MKL-8 derived from curry leaves due to the individuating bioactive potential that could offer more competitive opportunities related to therapeutic use, especially in alternative medicine or functional foods. *Lactococcus lactis* is a lactic acid bacterium that has been well described in dairy functions and is recognized to have probiotic capability and safety. Traditionally, it has been used as a food starter culture for dairy products and is approved by the Food and Drug Administration (FDA) as a GRAS^32^. The recent upsurge of resistance to antibiotics has increased the search for other forms of treatment. In this regard, *Lactococcus lactis*, one of the Lactic Acid Bacteria (LAB), has attracted attention because of its multifunctional properties. These properties, especially when obtained from non-dairy sources such as curry leaves, help in expanding the possibilities of *Lactococcus lactis* past the food fermentation industry, which may provide positive effects in chronic diseases and immune regulation. With growing bacterial resistance to conventional antibiotics, there is a growing focus on probiotics and the ways in which they can be used as natural remedies in medicine. *Lactococcus lactis* also has potent anti-inflammatory properties that make it play a critical role in damping inflammation related symptoms and regulating immune response^33^. Also, its anti-diabetic effect is a significant advantage because *Lactococcus lactis* has been reported to help maintain blood sugar stability and enhance the utilization of insulin^34^. These bioactivities indicate that *Lactococcus lactis* could be used as a promising and safer therapeutic approach compared to the conventional antibiotics for infections and chronic diseases. Specific antioxidant enzymes play a role in countering the effects of oxidative stress in LAB and the strain used in the current experiment, *Lactococcus lactis* MKL-8 exhibits significant amount of antioxidant activity. Concentration-dependent inhibition of free radicals was observed with MKL-8 being highly active with an inhibition of > 90. From this study, it is evident that MKL-8 has antioxidant activity and thus, may have potential health benefits especially in conditions where oxidative stress is apparent. 19% inhibition at 300 µl/ mL which supports the study done by Antolovich et al. (2002). Their study found LAB strain such as *Latilactobacillus curvatus* exhibiting high resistance to hydrogen peroxide^35^. In addition, our findings are in agreement with the previous study of Li et al., (2012) who also emphasized that LAB strains possess great antioxidant activity. *L. plantarum* strains obtained from Pineapple Puree were observed to possess substantial hydroxyl radical and DPPH radical scavenging activities; the highest activity of 90 ± 3% was however determined in *L. lactis* LL10. % to hydrogen peroxide^36^.

MKL-8 showed promising anti-diabetic efficacy by acting as α-amylase inhibitor with a maximum of 60% inhibition at 50 µg/ mL. This result is notable when compared with the 75.89% inhibitory potential recorded in *Lactobacillus brevis* KU15006^37^. Huang et al. (2015) also found that the α-amylase inhibition of the exopolysaccharides from L. plantarum H31 was different depending on the fractionation^38^. This suggests that the inhibitory activity of exopolysaccharides can be varied based on the structure and the extraction method highlighting the complexity of these biological activities. Muganga et al. (2015)^39^ identified that both the strains CCFM147 and CCFM240 have the ability to inhibit α-amylase with moderate efficiency of 32.9% and 27.9% respectively, indicating MKL-8 exhibits higher α-amylase inhibitory activity than these strains. A higher inhibitory activity of MKL-8 could be due to its distinct metabolic profile or the existence of molecules that would modulate the enzyme-inhibitory activity of the compound. Also, Alkalbani et al. in 2019 found that the water-soluble extract of the isolates KX881777, KX881772 and KX881779 have more than 34% activity against α-amylase^40^. Comparatively, the α-amylase inhibition by MKL-8 is 60% surpasses these findings, which implies its great potential of regulating enzyme activity connected with carbohydrate metabolism. The stronger inhibitory property of α-amylase of the MKL-8 suggests that this strain is well-suited in controlling blood glucose level because it can delay the rate of carbohydrate digestion which can be very helpful in preventing post-prandial hyperglycemia. Further, the inhibition levels of MKL-8 are also almost similar to the maximum inhibition recorded by Son et al. (2017) for L. brevis KU15006 being only 75.89%^41^. This supports the possibility of MKL-8 as an efficient α-amylase inhibitor which makes it a competitive strain in terms of anti-diabetic activity. This further underline the prospective of MKL-8 in controlling natural blood glucose level in accordance with the inhibitory impacts that have been observed in other probiotic strains.

One of the numerous known causes of inflammation and arthritic conditions is protein denaturation. Anti-inflammatory drugs, especially NSAIDs, have been documented to possess a dose-dependent ability to stabilize heat shock proteins, thus suppressing inflammation. These drugs may help to prevent protein from getting denatured, which is beneficial in treating various conditions^42,43^. In prevention of these effects, our study explores the effectiveness of MKL-8 in relieving inflammation by evaluating the effect of this protein in denaturation. Based on our observations, MKL-8 possess the ability to prevent protein denaturation, and this was substantiated by 99% inhibition at the highest concentration level of 300µL/ mL. This result is much higher than the positive control, Dexamethasone which inhibited 83% of the activity. This considerable inhibition implies that MKL-8 could be an effective in combating protein denaturation which is important in managing conditions that cause inflammation and tissue injury. It was also demonstrated that *Lactobacillus casei* and *Lactobacillus acidophilus* are prospective anti-inflammatory agents, like a significant enhancement in paw size in rats^44^. Carroll et al.^45^ examined the anti-inflammatory actions of MnSO4 liberated from *Lactobacillus gasseri* in a colitis mouse model. These studies also justify the use of *Lactobacillus* and *Lactococcus* species in the therapeutic management of inflammation. From both the strains it can be concluded that *Lactococcus* and *Lactobacillus* species may have potential to be explored further for anti-inflammatory therapies. Such significant suppression of protein denaturation implies that MKL-8 could potentially serve as an effective anti-inflammatory agent.

The biofilm formation by MKL8 strains is particularly significant for its potential use as a probiotic. The qualitative and quantitative analysis provided evidence that MKL8 possesses a strong biofilm-forming potential and synthesized EPS. The ability of MKL-8 to alter pathogenic biofilms also increases their potential benefits especially in the prevention of infections by antibiotic-resistant organisms. Such high levels of EPS synthesis indicate that the capacity for MKL8 to form biofilms is directly related to its ability to produce EPS, which is consistent with prior research on other probiotics such as *Lactobacillus fermentum*. In such studies, postbiotics, including EPS, reportedly influence biofilm formation through changing the interactions between microorganisms and the environment^46^. Though EPS play the role of biofilm formation in probiotics, they equally have the potency to degrade pathogenic biofilms. Jiang et al. (2011) noted that while EPS positively influences the formation of probiotic biofilms, there are difficulties in dealing with pathogenic biofilms in vivo. It is documented that pathogenic biofilms offer high resistance to antibiotics and the host immunity^47^. In line with this, our studies suggest that since MKL8’s EPS support its own biofilm formation, they may also enhance the probiotic efficiency by preventing pathogenic biofilms. In the gelatinase production assay, it was shown that MKL-8 did not produce gelatinase as there were no distinct zones seen on the gelatin plates as in the negative control. This means that MKL-8 does not produce gelatinase and thus does not possess gelatinase activity; this is in agreement with other studies where *Lactococcus lactis* for instance, was reported to lack gelatinase in vitro. Absence of gelatinase activity reinforces the conclusion about the avirulent character of MKL-8, as gelatinase is generally regarded as virulence factor in pathogenic strains. Thus, while some of the *Lactococcus* strains had genes homologous to the hemolysin-like proteins in their respective genomes, they have not exhibited signs of genuine virulence in actuality. Furthermore, although some *Lactococcus* possess gelatinase gene sequences, this activity has not been observed^48^. The overall effect of these results therefore supports the fact that MKL-8 is non-pathogenic adding more value to the fact that it could be a good candidate for a probiotic.

MKL8 crude metabolite extract derived from chloroform revealed several important bioactive secondary metabolites with GC-MS analysis. Out of all these, Oleic acid was identified and shown to possess immunomodulatory effects and can reduce cellular inflammatory and oxidative damage. Monounsaturated fatty acid, oleic acid, is relatively well-documented for its anti-inflammatory effects, which makes it a potentially useful compound for managing chronic inflammation and the diseases, including cardiovascular diseases. The involvement in modulation of immune responses and the antioxidant effect also suggest its therapeutic application. Also, Glycyl-L-Proline was detected for synthesizing collagen and helping to heal skin wounds that were also involved in this work^31^. Other compounds found are; 3-Azonia-5-Hexene-1-ol, N, N-Dimethyl-, Carbamate Ester, Bromide; Dichloroacetic Acid; 9-Eicosene (E); 2,5-Cyclohexadien-1-One, 3,5-Dihydroxy-4,4-Dimethyl-; and 3-(1,3,6- Trimethyl-4-Oxo-3-Piperidinyl) Propanenitrile. These compounds augment the hypothesis that MKL8 could be a rich source of novel bioactive molecules. Such observations provide support to the propound use of MKL8 as the probiotic candidate because they indicate great therapeutic potential in supporting the search for more bioactive molecules (Table 2). The presence of such diverse bioactive compounds support the notion that MKL8 could be further exploited for the development of a probiotic product which will not only have medicinal value but also functional food properties.

## Conclusion

The results of this study show that MKL-8 has high multifunctional probiotic potential. MKL-8 had significant free radical inhibitory and protein denaturation prevention abilities, and thus it could have potential in the regulation of oxidative stress and inflammation. Moreover, the strain had a very high percentage inhibition of α-amylase, thus possibly being used in diabetes management. Its ability to form biofilm and produce exopolysaccharides (EPS) also suggests its potential for gut colonization and inhibition of pathogenic microorganisms. The absence of gelatinase production is important, confirming that MKL-8 is non-pathogenic, a very important characteristic of a safe probiotic. GC-MS analysis identified bioactive compounds like oleic acid and glycyl-L-proline that contribute to the therapeutic effects of the strain, and FT-IR data was in agreement with these. In addressing the left-behind research gaps, this work is the first to isolate *Lactococcus lactis* from *Murraya koenigii* (curry leaf), exhibiting it as a potential probiotic with a safety advantage.It also emphasises on topics of current interest in probiotic research, namely strain safety and multifunctionality in food and health sector. In summary, the study highlights promising potential of MKL-8 as a probiotic strain, which holds significant health benefits particularly in managing oxidative stress, inflammation, and diabetes while ensuring safety through the absence of pathogenic traits.

## Data Availability

Sequence data that support the findings of this study have been deposited in the NCBI with the primary accession code OR342073.
